# Transfer of a Redox-Signal through the Cytosol by Redox-Dependent Microcompartmentation of Glycolytic Enzymes at Mitochondria and Actin Cytoskeleton

**DOI:** 10.3389/fpls.2012.00284

**Published:** 2013-01-09

**Authors:** Joanna Wojtera-Kwiczor, Felicitas Groß, Hans-Martin Leffers, Minhee Kang, Markus Schneider, Renate Scheibe

**Affiliations:** ^1^Department of Plant Physiology, Faculty of Biology and Chemistry, University of OsnabrueckOsnabrueck, Germany

**Keywords:** actin cytoskeleton, colocalization, glycolytic enzymes, microcompartmentation, mitochondria, redox-dependent binding, redox-signaling, VDAC

## Abstract

The cytosolic glyceraldehyde-3-phosphate dehydrogenase (GAPDH, EC 1.2.1.12, GapC) plays an important role in glycolysis by providing the cell with ATP and NADH. Interestingly, despite its glycolytic function in the cytosol, GAPDH was reported to possess additional non-glycolytic activities, correlating with its nuclear, or cytoskeletal localization in animal cells. In transiently transformed mesophyll protoplasts from *Arabidopsis thaliana* colocalization and interaction of the glycolytic enzymes with the mitochondria and with the actin cytoskeleton was visualized by confocal laser scanning microscopy (cLSM) using fluorescent protein fusions and by bimolecular fluorescence complementation, respectively. Yeast two-hybrid screens, dot-blot overlay assays, and co-sedimentation assays were used to identify potential protein–protein interactions between two cytosolic GAPDH isoforms (GapC1, At3g04120; GapC2, At1g13440) from *A. thaliana* with the neighboring glycolytic enzyme, fructose 1,6-bisphosphate aldolase (FBA6, At2g36460), the mitochondrial porin (VDAC3; At5g15090), and actin *in vitro*. From these experiments, a mitochondrial association is suggested for both glycolytic enzymes, GAPDH and aldolase, which appear to bind to the outer mitochondrial membrane, in a redox-dependent manner. In addition, both glycolytic enzymes were found to bind to F-actin in co-sedimentation assays, and lead to bundling of purified rabbit actin, as visualized by cLSM. Actin-binding and bundling occurred reversibly under oxidizing conditions. We speculate that such dynamic formation of microcompartments is part of a redox-dependent retrograde signal transduction network for adaptation upon oxidative stress.

## Introduction

The glycolytic pathway consists of 10 enzymes that catalyze the reversible oxidation of glucose to pyruvate with generation of ATP and a reductant (NADH), and provides pyruvate for plant mitochondrial respiration. Glycolysis supplies also carbon skeletons for other biosynthetic processes, such as synthesis of fatty acids, nucleic acids, isoprenoids, and amino acids, being therefore important in actively growing autotrophic tissues. Moreover, glycolysis becomes a crucial player in many biochemical adaptations to environmental stresses such as nutrient limitation, osmotic stress, drought, hypoxia, anaerobiosis, and cold/freezing, as well as during seed germination (Plaxton, 1996). Since these aspects are crucial for plant development and growth, there has to be a multi-faceted regulation of glycolysis in plants. In particular, the posttranslational modifications of plant glycolytic enzymes may be of key importance upon oxidative/nitrosative stress (Dixon et al., 2005; Lindermayr et al., 2005), since S-nitrosylation of the *Arabidopsis* GAPDH, for instance, was shown to inhibit its activity in a reversible manner (Lindermayr et al., 2005). Similar effects were demonstrated for the GSSG- and GSNO-treated recombinant cytosolic GAPDH (Holtgrefe et al., 2008), and for cytosolic aldolase from *Arabidopsis thaliana* (van der Linde et al., 2011), as well as for the S-glutathionylated triose-phosphate isomerase (Ito et al., 2003). Nuclear localization was also reported for the glycolytic isoenzymes from the cytosol (Hameister et al., 2007; Holtgrefe et al., 2008; van der Linde et al., 2011).

Another regulatory aspect influencing the glycolytic pathway seems to be its spatial organization in the plant cell (Fernie et al., 2004). Change of the cellular microenvironment may trigger new effects, i.e., transient protein–protein interactions, formation of a metabolon, protein association with certain subcellular structures, such as organelle membranes or cytoskeletal lattice, or translocation to other subcellular compartments. Observations made mainly in animal cells, and only recently in plants, gave hints for variable subcellular localizations of certain glycolytic enzymes that were classically considered as a soluble system of proteins. A long list of glycolytic enzymes associated with the cytoskeleton in animal cells accumulated over the past decades (Walsh et al., 1980, 1989; Somers et al., 1990; Schindler et al., 2001; Schmitz and Bereiter-Hahn, 2002), but the cytoskeleton-association phenomenon has been reported for plant glycolytic enzymes only in the last years (Azama et al., 2003; Holtgräwe et al., 2005; Balasubramanian et al., 2007).

In our previous observations, the *Arabidopsis* mesophyll cells, transiently expressing a GFP-fusion with GapC and aldolase isoforms, respectively, were found not only to exhibit cytosolic and nuclear fluorescent signals (Holtgrefe et al., 2008; van der Linde et al., 2011), but signals also occurred as foci-like structures of yet unknown nature. Moreover, in our earlier work, a mitochondrial porin, VDAC1a, had been identified as a putative binding partner in a yeast two-hybrid screen of a maize seedling cDNA library (Holtgräwe et al., 2005). Hence, in the light of recent reports on possible mitochondrial microcompartmentation of several glycolytic enzymes (Giegé et al., 2003; Holtgräwe et al., 2005; Kim et al., 2006; Balasubramanian et al., 2007; Damari-Weissler et al., 2007; Graham et al., 2007), the observed fluorescent GapC1 and GapC2 foci were further analyzed in the context of their association with organelles and cytoskeleton. Therefore, Bimolecular Fluorescence Complementation (BiFC), the “one-on-one” version of the yeast two-hybrid assay, dot-blot overlay assays, and co-sedimentation assays with F-actin were applied to test the possible interactions between both *Arabidopsis* GapC isoforms, aldolase, VDAC3, and the actin cytoskeleton. Considering the glycolytic enzymes, it became a challenge to reveal their spatial organization, since it seems to play a regulatory role (Fernie et al., 2004). However, little is known about factors influencing the dynamic microcompartmentation of glycolytic enzymes *in planta* (Graham et al., 2007). Transient changes are possibly triggered by redox-changes occurring in the cytosol, when the cells are exposed to stress. They might serve as a signal, leading to reorganization of the cytosol, and finally to changed cellular functions and acclimation. The *in vitro* studies presented here were performed in order to test redox-dependency of the protein–protein interactions between glycolytic enzymes, actin cytoskeleton and the outer mitochondrial membrane (OMM) through VDAC.

## Experimental Procedures

### Isolation of mesophyll protoplasts from *A. thaliana* plants

Mesophyll protoplasts isolated from of *A. thaliana* plants (ecotype Columbia) were used for *in vivo* labeling of the subcellular structures and organelles, such as Golgi apparatus, nucleus, actin cytoskeleton, and mitochondria, as well as visualization of subcellular localization of proteins fused to CFP, GFP, or YFP. Protoplasts were isolated from leaves of 5- to 6-week-old wild-type *A. thaliana* plants, according to Seidel et al. (2004), with some modifications (Voss et al., 2008).

### Visualization of fluorescence-tagged proteins in transiently transformed protoplasts from *A. thaliana*

The glycolytic enzymes were expressed in *Arabidopsis* protoplasts from the pGFP-2 vector as a C-terminal fusion with the Green Fluorescent Protein (pGFP GapC1 and pGFP GapC2), under control of the constitutive CaMV 35S promoter (Kost et al., 1998). Similar experiments were carried out with vectors encoding GapC isoforms as CFP fusions, based on the p-35S-CFP-NosT vector, which was kindly provided by Thorsten Seidel (University of Bielefeld, Germany). A chimera of the mCherry protein with a transmembrane domain of a rat α-2,6-sialyl-transferase, which is a mammalian, Golgi-targeted glycosylation enzyme (Saint-Jore et al., 2002), was used in order to visualize Golgi apparatus in the plant cell. This construct was obtained from Ekkehard Neuhaus (TU Kaiserslautern, Germany). Visualization of actin microfilaments in plant cells was performed using a vector encoding tdTomato: AtFim1 ABD2, kindly provided by Takumi Higaki (University of Tokyo, Japan; Sano et al., 2005). It consists of the second actin-binding domain (ABD2) of the fimbrin-like protein from *A. thaliana* (AtFim1), fused with tdTomato (Shaner et al., 2004). Plant mitochondria were visualized by staining mesophyll protoplasts with the MitoTracker^®^ Orange CMTMRos (Molecular Probes/Invitrogen, Karlsruhe, Germany).

The protein–protein interactions between the glycolytic enzymes, GapC and aldolase, with VDAC3, were investigated by means of the BiFC technique (BiFC or split YFP; Hu et al., 2002). Appropriate vectors, pUC-SPYNE and pUC-SPYCE (Walter et al., 2004) obtained from Prof. Jörg Kudla (University of Muenster), were used to design constructs expressing the respective proteins. Fusions of aldolase (FBA6, At2g36460), VDAC3 (At5g15090), or GapC1 and GapC2 with the N-terminal or the C-terminal half of the YFP are described as X:YFP^N^ or X:YFP^C^, respectively. Combination of plasmids, pUC-SPYNE-bZip63 and pUC-SPYCE-bZip63, coding for an YFP-fusion with the transcription factor bZip63, was used as a positive control in the BiFC assay. Homodimerization of this protein was already demonstrated to occur in the plant nucleus (Walter et al., 2004).

The transiently transformed protoplasts incubated between 8 and 16 h, were stained with 50 nM MitoTracker^®^ Orange CMTMRos for 15 min at RT, in the dark, prior to imaging. Afterwards, the sample was centrifuged for 1 min at 100 × *g*, and the resulting pellet was resuspended with 1 ml W5 solution. The plant cells were immediately observed using the cLSM 510 META (Carl Zeiss, Göttingen, Germany). CFP and autofluorescence of chlorophyll were visualized with excitation at 458 nm and emission at 480–520 and 650–710 nm, respectively. GFP and autofluorescence of chlorophyll were visualized with excitation at 488 nm and emission at 500–530 nm and 650–710 nm, respectively. YFP and autofluorescence of chlorophyll were visualized with excitation at 514 nm and emission at 535–590 nm and 650–704 nm (detected with META Detector), respectively.

When observed with stained mitochondria, GFP and autofluorescence of chlorophyll were visualized with excitation at 488 nm and emission at 500–530 nm and 650–704 nm, respectively, whereas MitoTracker^®^ Orange was excited with 543 nm and emitted at 565–615 nm. The same settings were used for the combination with GFP, when actin cytoskeleton was additionally visualized. In case, when YFP was expressed in *Arabidopsis* protoplasts along with stained mitochondria, YFP was visualized with excitation at 514 nm and emission at 522–533 nm, whereas MitoTracker^®^ Orange was excited with 543 nm and emitted at 565–597 nm, both detected with META Detector, respectively.

### Yeast two-hybrid screen with GapC1 and 2 as baits, and “one-on-one” assays

The two isoforms of GAPDH from *A. thaliana* (GapC1, At3g04120) and GapC2, At1g13440) were applied as baits in the two-hybrid screen of the cDNA library for positive protein–protein interactions, according to Kolonin et al. (2000) with small modifications. The cDNA library from *A. thaliana* suspension cell culture was obtained from Prof. Koncz, University of Bonn (Nemeth et al., 1998). Screens were performed according to manufacturer’s instructions (The Matchmaker Two-Hybrid Library Construction and Screening Kit, Clontech). As further confirmation of the interactions found in the DNA-library screen, the putatively positive interactions were tested in a “one-on-one” assay. For this aim, the haploid AH109 strain provides all needed reporter genes (HIS3, ADE2, MEL1), so that it could be cotransformed with both vectors encoding the investigated bait and prey proteins. The positively transformed yeast colonies, with two replicates in each case, were tested for activation of the reporter genes ADE2, HIS3, MEL1 by “drop test” on selective media lacking tryptophane, leucine, and histidine (SD/TDO). For strong protein–protein interaction, medium was additionally lacking adenine (SD/QDO/X-α-Gal). In case of control experiments, yeast cells were transformed with a combination of the investigated partner and empty bait (pGBKT7) or prey vector (pGADT7).

### Co-sedimentation assay

Since plant and animal actin are highly conserved in sequence (88% identity), co-sedimentation experiments were performed with commercially available rabbit muscle actin (>99% pure) that was purchased from Cytoskeleton (Denver, CO, USA). Actin was purified by a polymerization/depolymerization cycle prior to usage. In order to exclude the possibility to capture soluble enzymes during polymerization without specific binding, the polymerization step was performed prior to the addition of the soluble enzymes.

G-actin was prepared in low-salt buffer (LSB; 5 mM Tris-HCl pH 7.6, 0.2 mM CaCl_2_, 0.2 mM ATP, 0.15 mM NAD) by incubation of rabbit muscle actin for 30 min on ice, buffer change over Pierce Desalting Spin Columns (Rockford, USA) and ultracentrifugation at 100,000 × *g* for 1 h at 4°C. G-actin was polymerized upon addition of 1x polymerization inducer [PI; 50x stock: 250 mM Tris-HCl (pH 7.6), 100 mM MgCl_2_, 50 mM ATP, 7.5 mM NAD, 2 M KCl] at a protein concentration of 0.4 mg/ml in LSB. Aldolase (FBA6, At2g36460) GAPDH (GapC1; At3g04120), and BSA (negative control) were also adjusted to a protein concentration of 0.4 mg/ml with 1x assay buffer [50x stock: 250 mM Tris-HCl (pH 7.6), 100 mM MgCl_2_, 50 mM ATP, 7.5 mM NAD]. F-actin and the glycolytic protein/s were mixed with a 1:1 ratio on a protein basis, and redox reagents (final concentrations: diamide + GSH: each 1 mM, GSSG: 5 mM, GSNO: 0.5 mM, H_2_O_2_ + GSH: each 0.5 mM) were added. The mixtures were incubated at 22°C for 30 min. Then DTT_red_ (final concentration 10 mM) was added to one part of the mixtures, followed by incubation for 5 min at 22°C. Then supernatant and pellet were separated by ultracentrifugation at 100,000 × *g* for 1 h at 22°C. Both fractions were analyzed by SDS-PAGE (10%) and subsequent staining of the gels with Coomassie Brilliant Blue R.

### *In vitro* visualization of actin

Samples of F-actin together with GapC1, aldolase, or both enzymes were prepared as described for the co-sedimentation assay. Then DTT_red_ (10 mM final concentration) was added to the mixtures for another incubation at 22°C for 5 min and stained with phalloidin labeled with tetramethylrhodamine B isothiocyanate (Sigma-Aldrich; final concentration 70 nM). Fluorescence was visualized by cLSM (510 META, Zeiss, Jena, Germany) using the Plan-Apochromat 63x/1,4 Oil DIC objective. Picture calculation and processing were done with the LSM Image Browser (Zeiss, Jena, Germany).

### Dot-blot overlay assay with crosslinking

Overlay dot-blot analyses were performed with recombinant GAPDH; GapC1, At3g04120, VDAC3 (At5g150910) reconstituted into liposomes, purified F-actin, G-actin (from rabbit muscle, Sigma-Aldrich), and BSA as a negative control. In the case of VDAC3, TOM40, and empty liposomes were used as negative controls. The proteins (each 2 μg) were dripped onto nitrocellulose membranes and air-dried. The membranes were blocked with 6% BSA in TBS-T (50 mM Tris-HCl, 150 mM NaCl, and 0.2% Tween 20, pH 8) for 1 h and washed. Membranes were then incubated with 100 μg/ml recombinant cytosolic fructose 1,6-bisphosphate aldolase (FBA6, At2g36460) in the presence of 10 mM DTT, 0.1 mM GSNO, 5 mM GSSG, 1 mM diamide plus 1 mM GSH or 0.5 mM H_2_O_2_ plus 0.5 mM GSH, or VDAC3 in liposomes, respectively, for 1 h at room temperature and finally washed. For cross-linking, the membranes were treated with 0.115% glutaraldehyde in 20 mM HEPES buffer (pH 7.5) for 5 min at 37°C and washed (Migneault et al., 2004). Immunodetection was achieved with polyclonal antibody against maize aldolase (1:10,000) or against VDAC1 (1:5000, Agrisera, Sweden) for 1 h at room temperature and color development using goat anti-rabbit IgG conjugated with alkaline phosphatase (1:5000; BioRad, Munich) using BCIP/NBT as substrates, or the second antiserum conjugated with horseradish peroxidase (1:3000, BioRad, Munich) using ECL (iNtRON Biotechnol., Korea) as substrate.

For the dot-blot analyses, VDAC3 was synthesized in a cell-free system (RTSTM 100 Wheat Germ CECF, 5PRIME, Hamburg, Germany) and separated from the reaction mix via reconstitution into liposomes in the presence of 80 mM nonanoyl-*N*-methylglucamide (MEGA 9). The reconstitution of VDAC3 into liposomes was verified by a Nycodenz^®^ density gradient flotation assay of the proteoliposomes. The dot-blot assay was performed as described above in the presence and the absence of 10 mM DTT.

### Homology modeling of 3D-structures

The three-dimensional structures of the proteins were generated using the homology modeling mode of the SWISS-model workspace (Bordoli et al., 2009). The template structures for the homology modeling of the *A. thaliana* proteins were selected in accordance with the protein structures which were used by Forlemu et al. (2011). For the comparison of the amino acids which were identified by Forlemu et al. (2011) and Ouporov et al. (1999) to be involved in the ionic interaction, a multiple sequence alignment was performed.

## Results

### *In vivo* colocalization of GapC with mitochondria

**Figure 1:**
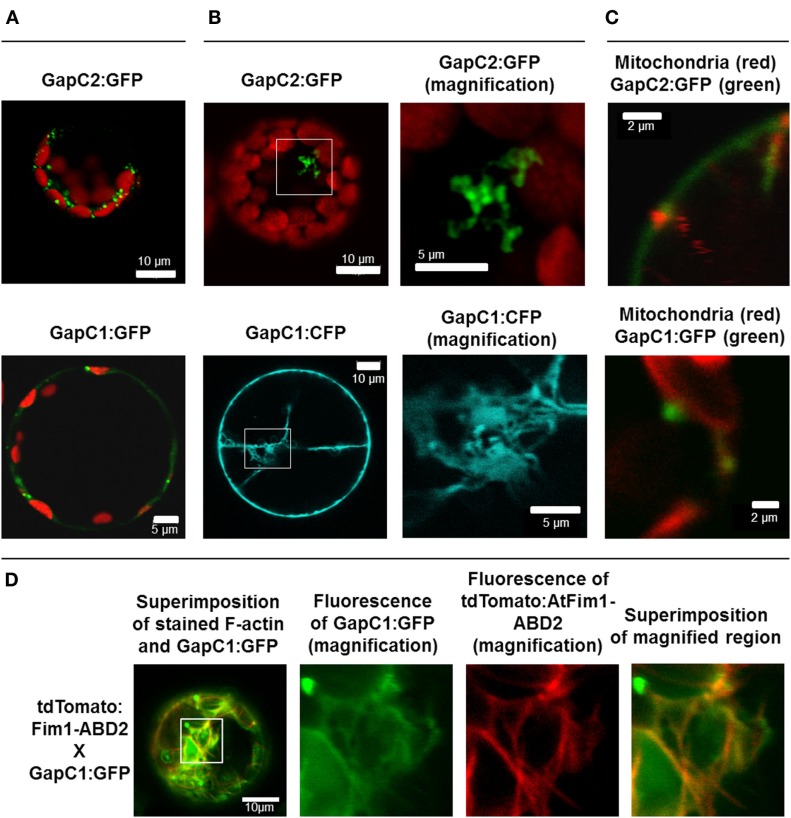
**Manifold localization patterns of the GAPDH isoforms**. GFP- and CFP fusions of the two isoforms of GAPDH (GapC1 and GapC2) were transiently expressed in the protoplasts, isolated from leaves of 6-week-old plants of *A. thaliana*. **(A)** Superimposition of chlorophyll autofluorescence and locally accumulated GFP-fusions of the two isoforms. **(B)** GFP- and CFP-fluorescence from the GapC1 and GapC2 fusions, respectively. On the right hand side, magnifications are shown of relevant parts. **(C)** Colocalization of GFP-fused GapC1 and GapC2 with MitoTracker-stained mitochondria. **(A–C)**. Pictures in the last lane present the magnified areas indicated in the overlays. Images in the YFP and MitoTracker channel were taken in the frame mode, both channels separately which caused a short time delay between the red and green channel, due to respective dichroic mirror settings. **(D)** Protoplasts were transformed with GapC1:GFP (green) and tdTomato:Fim1ABD2 (red). The fotos in the panels 2, 3, and 4 show the magnification of the boxed area in foto 1. All images were taken with the confocal Laser Scanning Microscope LSM 510 META, Zeiss.

Apart from the even cytosolic distribution and the occasional nuclear localization of the GFP-fused GapC1, GapC2, and aldolase, reported previously (Holtgrefe et al., 2008; van der Linde et al., 2011), fluorescent signals appeared in a non-homogeneous distribution as locally accumulated foci of GapC2:GFP and GapC1:GFP/CFP in the cytosol of the transformed protoplasts (Figures 1A,B). The GapC-containing structures emitted intense fluorescence that in many cases was much stronger than the cytosolically distributed GFP-fused enzyme, which was frequently observed. The diffuse cytosolic signal was therefore not imaged in certain cases, due to down-regulation of the detector in the cLSM 510 META (Figure 1, GapC2:GFP). The cytosolic aggregates of GapC:GFP seemed to vary with regard of their size, shape, and amount. A higher magnification resulted in a better resolution of the foci-like spots, showing their branched constitution in several cases (Figure 1B, magnified GapC2:GFP) and suggested that these fluorescent structures, formed by GapC:GFP isoforms, could be associated with certain organelles.

Interestingly, the presence of At1g13440- and At3g04120-encoded GapC in the secretory pathways was predicted using the ARAMEMNON database (data not shown) and was reported by Santoni et al. (1998) and Marmagne et al. (2004), who both identified GapC in the PM protein fraction from *Arabidopsis*. In addition to PM, vacuole, endoplasmic reticulum (ER), and the Golgi apparatus constitute the major components of the plant secretory system (Brandizzi et al., 2004). In this context, the fraction of manifold, small GapC:GFP foci, resembling plant Golgi apparatus, was tested for colocalization with Golgi stacks using a chimera of the mCherry protein with a transmembrane domain of a rat α-2,6 sialyl-transferase (Saint-Jore et al., 2002). However, no colocalization of signals emitted by GapC:GFP foci and by the Golgi apparatus could be detected (data not shown).

**Figure A1:**
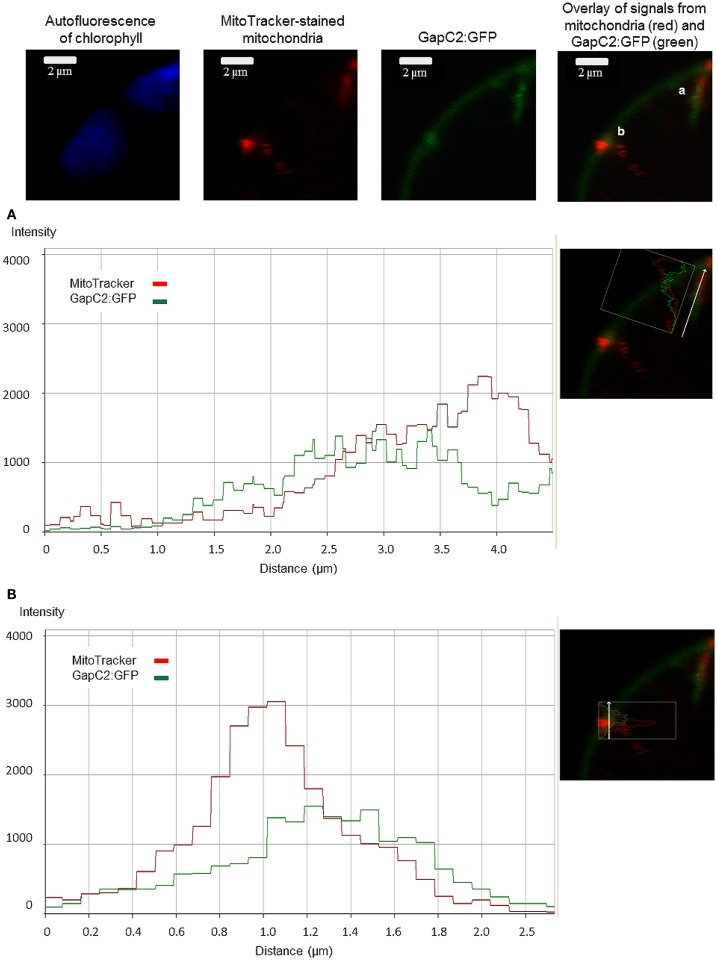
**Colocalization analysis of GAPDH (GapC2) with mitochondria**. The magnified section of the protoplast expressing GapC2:GFP displays regions of a possible association of the glycolytic enzyme with the mitochondrion, stained with 50 nM MitoTracker^®^ Orange CMTMRos (Molecular Probes). The diagrams show the colocalization analysis using fluorescence-intensity profiles of both fluorescent signals **(A,B)**.

**Figure A2:**
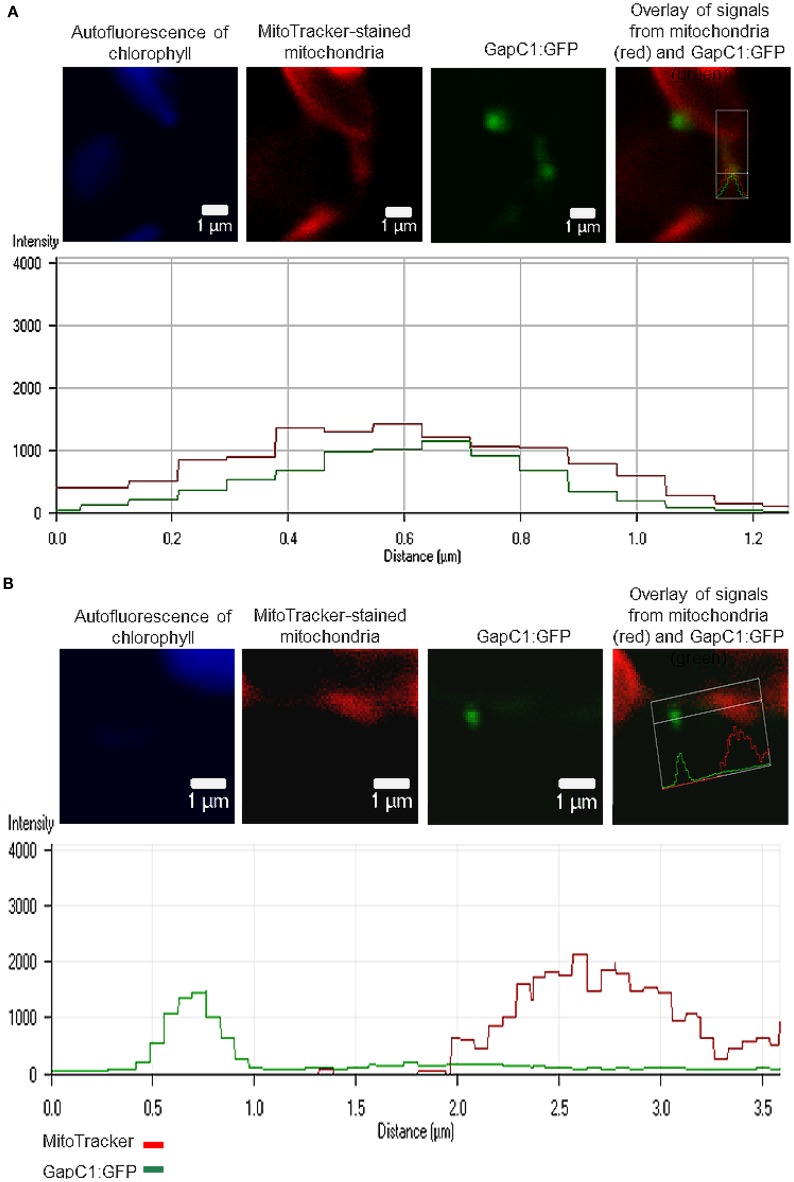
**Occasional colocalization of GAPDH (GapC1) with mitochondria**. GapC1:GFP was transiently expressed in the protoplasts, isolated from Arabidopsis plants. Mitochondria were stained using 50 nMMitoTracker^®^ Orange CMTMRos (Molecular Probes). Two regions of interest (ROI), depicted with a white frame, were magnified in order to perform a colocalization analysis by means of a fluorescence-intensity profile. One ROI displays a possible association of the glycolytic enzyme with mitochondrion **(A)**, whereas the other ROI shows no mitochondria association at the moment of visualization **(B)**.

GapC-organelle association was then tested with the mitochondria dye MitoTracker^®^ Orange. Fluorescence-intensity profiles of the signals emitted by both GFP-fusion GapC and MitoTracker^®^ Orange enabled a colocalization analysis, which indicated that some of the smaller foci of GapC:GFP were associated with mitochondria (Figure 1C; Figures A1 and A2 in Appendix), whereas the bigger branched structures were never close to these organelles (data not shown).

**Figure 2:**
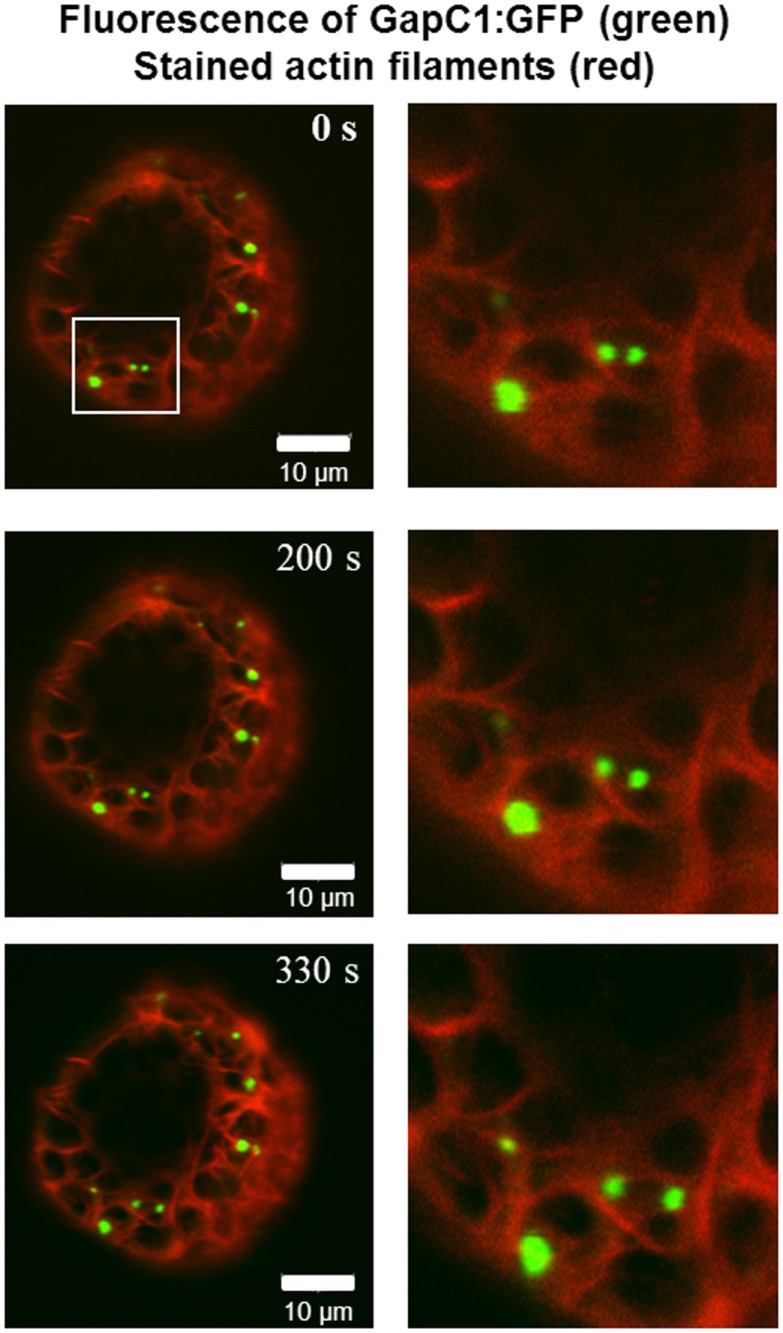
**Time-lapse images of GapC1:GFP aggregates with respect to the actin cytoskeleton**. Time-lapse images of the GAPDH aggregates in relation to the actin cytoskeleton show GapC1:GFP aggregates moving along actin fibers (depicted with white frames). Images were taken after the indicated times. Actin filaments are stained with tdTomato:AtFim1 ABD2 (red). The GapC1:GFP foci are displayed in green. Scale bar: 10 μm. The images were taken with the Confocal Laser Scanning Microscope LSM 510 META, Zeiss.

**Figure A3:**
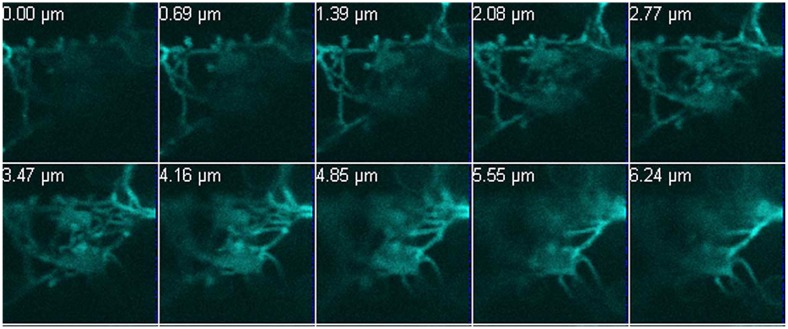
**A network formed by the fluorescently labelled GAPDH**. A 13-μm deep Z-stack was performed to demonstrate the filamentous structures of GapC1:CFP in three dimensions in the examined protoplast. The appropriate scale bar is included in the Figure 1. The images were taken with the Confocal Laser Scanning Microscope LSM 510 META, Zeiss.

**Figure A4:**
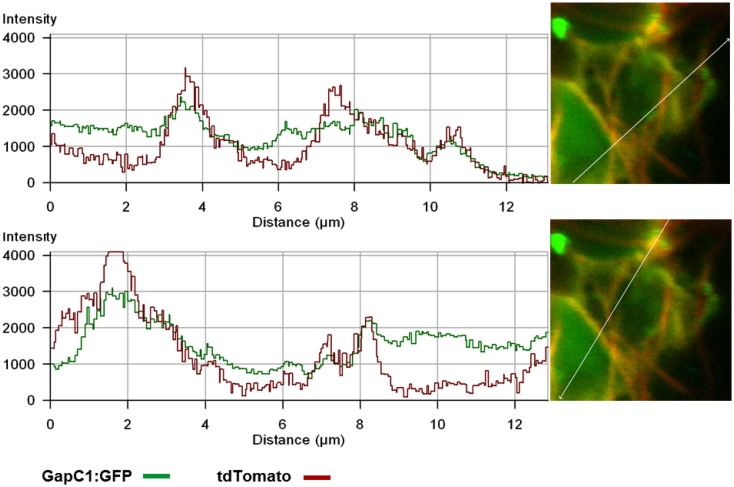
**Overlap of fluorescence emission from tdTomato-Fimbrin and GapC-GFP**. Fluorescent signals of GapC1:GFP and F-actin, stained with tdTomato:AtFim1-ABD2, were analyzed with the fluorescence-intensity profile. The GapC1:GFP protein seems to bind the stained actin filaments in the plant cell, since the plotted fluorescent signals partially cover the same regions along a distance of approximately 12 μm. The diagram was performed with the Zeiss LSM 5 software.

GapC isoforms can also form cytoplasmic strands when expressed transiently as CFP fusions that might be correlated with cytoskeleton (Figure 1B, lower pictures, Figure A3 in Appendix). When fluorescent fusion of tdTomato with the actin-binding domain ABD2 of fimbrin (AtFim1) was applied for staining actin filaments, an additional peak for tdTomato, that appeared within the GFP-emission range, was noticed in protoplasts that were transiently expressing GapC1:GFP (Figure 1D; Figure A4 in Appendix). Therefore, the analysis of cytoskeletal association of GapC isoforms had to be modified. During microscopic imaging with cLSM 510 META it turned out that, by down-regulation of the detector, the bleeding-through artifacts could be removed, allowing visualization of the stronger GapC1:GFP foci only, with respect to the stained actin cytoskeleton. This approach enabled the observation of the punctuate signals emitted by GFP-fusion GapC, and showed that the clusters might move between actin fibrils, but their direct association with stained actin filaments was not obvious (Figure 2).

### Protein–protein interaction partners of GapC in the yeast-2-hybrid system

Identification of new interaction partners of GapC by a yeast two-hybrid screen of a cDNA library could elucidate its variable subcellular localization and novel “moonlighting” features, which have been suggested for the animal counterpart (for review see: Sirover, 1999, and Sirover, 2005). Such approach might also help to reveal, why this cytosolic enzyme can localize to different subcellular structures, such as nucleus (Holtgrefe et al., 2008), or possibly with the OMM, which has been recently observed (Giegé et al., 2003; Graham et al., 2007), and what we follow in this study. Here, a yeast two-hybrid screen of the cDNA library from *A. thaliana* was performed. Among 42 putative interaction partners of the GapC2, four cDNA coding for the voltage-dependent anion channel VDAC3 (At5g15090), a porin from the OMM, and 11 GapC clones could be identified. The presence of yeast colonies expressing GapC1 and GapC2 protein on SD/QDO/X-α-Gal is a hint to suggest positive, but weaker interactions between subunits of different GapC isoforms, which form not only homo-, but also heterooligomers. A yeast two-hybrid screen with GapC1 as bait protein revealed 16 protein–protein interaction partners of this isoform. Among them, 13 sequences were full-length clones encoding VDAC3 (At5g15090).

**Figure 3:**
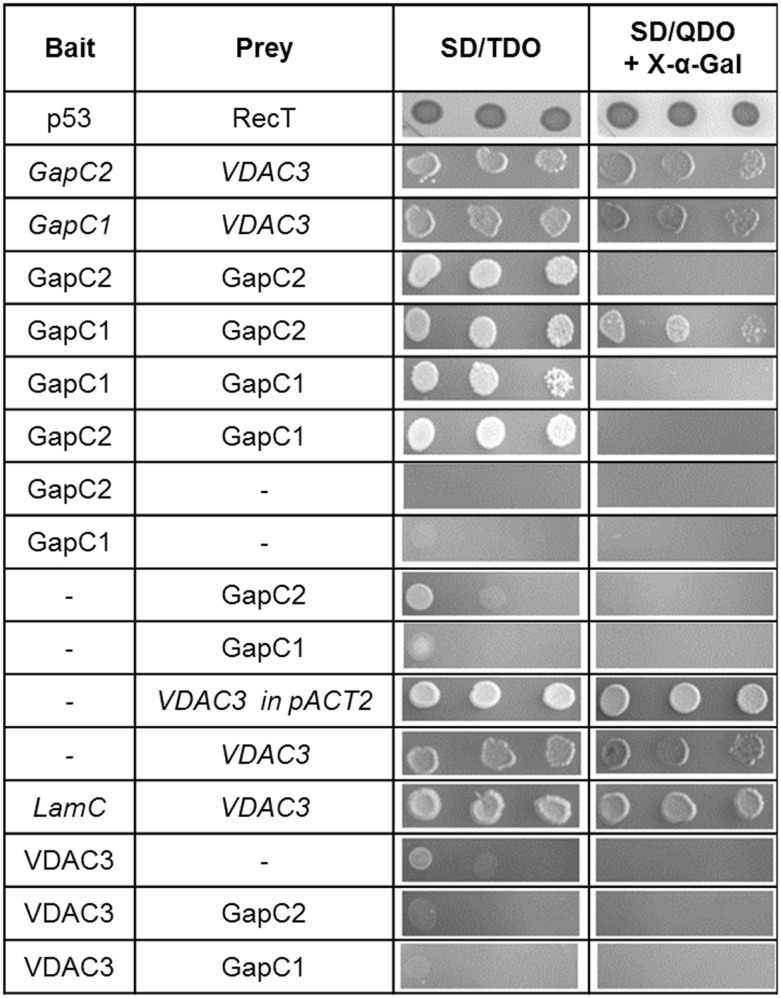
**Yeast 2-hybrid “one-on-one” assays between GapC isoforms, aldolase, and VDAC3**. Putatively positive interactions had been identified in the yeast two-hybrid screen of the cDNA library from *A. thaliana*. Here, to retest these findings, the haploid yeast strain AH109 was cotransformed with constructs, expressing bait, or prey proteins, as indicated in the table. Serial dilution of the appropriate overnight yeast culture was dropped onto selective media to demonstrate the stringency of binding between proteins. Colonies growing on SD/Leu^−^, Trp^−^ were successfully transformed with both vectors, encoding the investigated proteins. Growth on SD/TDO and SD/QDO/X-α-Gal is a sign of positive interactions. Italics indicate false positive results due to autoactivation by VDAC3 when used as a prey.

Both isoforms, GapC1 and GapC2, and VDAC3 were applied in an additional set of “one-on-one” yeast two-hybrid assays, in order to retest the specificity of these associations. The colonies containing hybrid GapC isoforms fused with the GAL4-BD domain, and VDAC3 fused with the GAL4-AD domain grew under high-stringency conditions, confirming the positive interaction found in the yeast two-hybrid screen (Figure 3). With help of control vectors, it was surprisingly shown that autoactivation of all reporter genes took place in the case of VDAC3-AD (expressed from the pACT2 or pGADT7 vector), cotransfected with an empty vector pGBKT7 or pGBKT7-LamC. The appropriate yeast colonies grew on SD/TDO and additionally on SD/QDO/X-α-Gal. This unspecific autoactivation of reporter genes seemed to be a property of VDAC3-AD, which was not the case for the VDAC3-BD, expressed from pGBKT7 under a truncated ADH1 promoter (Figure 3). However, it also did not interact with any of the GapC isoforms. The initially identified VDAC3-AD and GapC-BD interaction could therefore not be confirmed, due to the autoactivating property of VDAC3-AD. Excluding the effect of autoactivation by only using VDAC3 as a bait, the interaction with aldolase was also tested. Surprisingly, only a weak interaction of aldolase with itself when forming an homooligomer was found. Its interactions with GapC1 and 2, or with VDAC3 were negative in this assay (data not shown).

### Bimolecular fluorescence complementation approach to test the *in vivo* interaction of GapC and aldolase with mitochondrial porin

**Figure 4:**
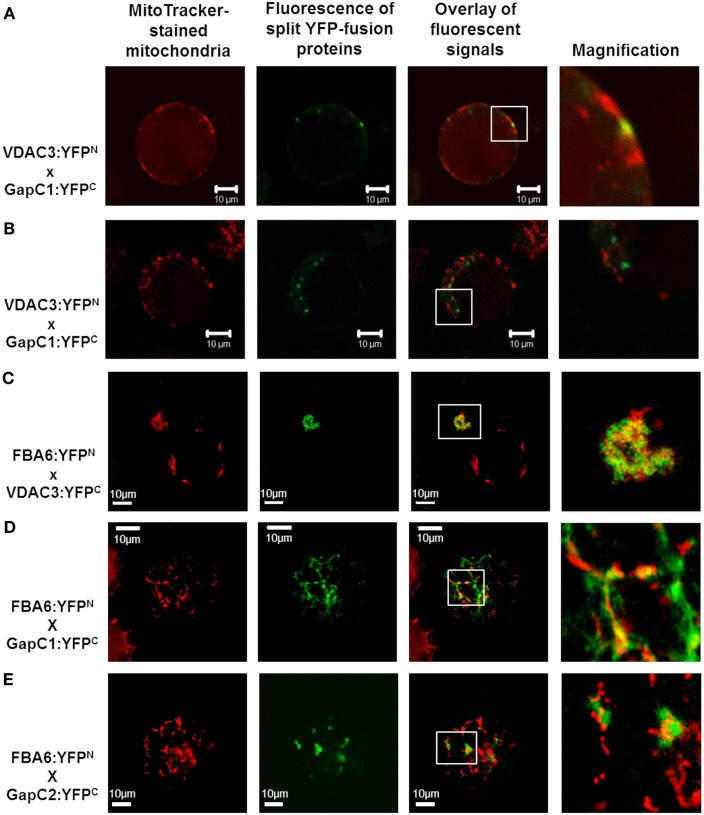
**Colocalization of aldolase (FBA6) and GapC with mitochondria**. Vectors encoding the investigated proteins were cotransformed in protoplasts, isolated from leaves of *A. thaliana* plants. The fluorescent signals from GapC1 interacting with VDAC3 **(A,B)**, FBA6, and VDAC3 **(C)**, as well as FBA6 with GapC1 or GapC2 **(D,E)**, fused with N- or C-terminal halves of YFP, respectively, were tested for mitochondrial localization by staining the cells with 50 nM MitoTracker^®^ Orange CMTMRos (Molecular Probes).

Split YFP fusions of GapC isoforms and VDAC3 were expressed in *Arabidopsis* protoplasts, with the aim to verify their putatively direct interaction *in planta*, which was found in the yeast two-hybrid screen, but appeared to be false positive in the “one-on-one assay” (Figure 3). In both combinations of  YFP fusions, fluorescent signals could be detected in protoplasts. Some of the fluorescent foci were colocalized with mitochondria (Figure 4A), but other observed signals, reflecting interaction between GapC and porin, did not overlap with the organelles stained with MitoTracker^®^ Orange (Figure 4B), which was also the case for GapC:GFP (Figure 1, Figures A1 and A2 in Appendix).

**Figure A5:**
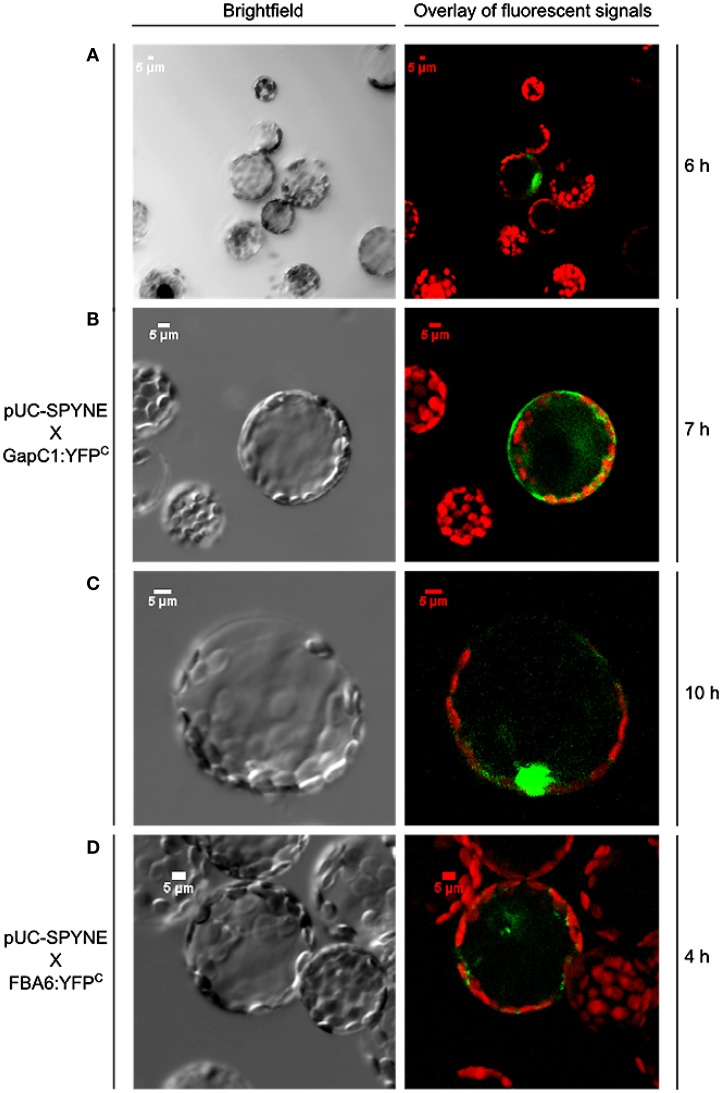
**Negative controls for BiFC experiments analyzing any colocalization of aldolase (FBA6) and GapC1**. Vectors encoding the investigated proteins were cotransformed in protoplasts, isolated from leaves of A. thaliana plants. **(A-C)** is a time course of the expression of the empty vector with only the N-terminal half of YFP and GapC1 fused with the C-terminal half of YFP. In **(D)**, the same is shown for FBA6 after 4 h.

Previous screening of the cDNA library from hypoxic seedlings of *Zea mays*, as well as a dot-blot overlay assay had demonstrated binding of the cytosolic isoform of maize fructose 1,6-bisphosphate aldolase to the mitochondrial porin, VDAC1a (Holtgräwe et al., 2005). It was therefore tempting to look also at this interaction *in vivo*, in *Arabidopsis* protoplasts, that enabled the expression of aldolase fused to the N-terminal (FBA:YFP^N^), and VDAC3 fused to the C-terminal half of the YFP (VDAC3:YFP^C^). Indeed, fluorescent signals occurred in a locally accumulated form. Additional staining of mitochondria confirmed the location of the fluorescent foci next to these organelles (Figure 4C). No signals were observed, however, when vectors encoding aldolase fused to the C-terminal (FBA:YFP^C^), and VDAC3 fused to the N-terminal half of the YFP (VDAC3:YFP^N^) were used. Therefore, sterical factors may determine whether the direct binding of aldolase to VDAC3 can occur. This localization pattern was not detected in the negative controls (Figure A5 in Appendix). When an empty pUC-SPYNE vector and GapC1, GapC2, or FBA6, fused to the C-terminal halves of the YFP, were used, positive BiFC signals that were homogeneously distributed in the cytoplasm or even localized in the nucleus, were surprisingly found in several protoplasts. An identical, cytosolic localization pattern was observed also in the *Arabidopsis* protoplasts expressing split YFP-fused GapC1 and/or GapC2, reflecting the homo- and heterooligomerization of its subunits, respectively (data not shown). Beside this cytosolic compartmentation, FBA6 and GapC interacted also in a locally accumulated way, in some cases close to the stained mitochondria (Figures 4D,E), which correlated with the microscopic observations regarding potential mitochondrial association of GapC:GFP (Figures 1 and 2; Figure A1 in Appendix). With respect to the negative controls that were false positive, the cytosolic, YFP-like fluorescent signals are not easily distinguishable from the false positive interaction. These putatively positive results may be unspecific and the homogeneous distribution of interacting glycolytic enzymes could be artifactual. The irreversible nature of the association between the two YFP fragments, especially when they are overexpressed or documented after a longer period of the transient expression, namely over 24 h, may be the reason for the false positive signals (Zhong et al., 2008).

### *In vitro* interactions between GAPDH, aldolase, rabbit actin, and VDAC and their redox-dependence

From the above described experimental approaches that are frequently used to study protein–protein interactions, no uniform picture could be drawn as to the *in vivo* binding of soluble glycolytic enzymes to cellular structures. This is probably due to the fact that the cellular state and the actual microenvironment of the interacting partners cannot easily be controlled. Unknown cellular factors, such as posttranslational modifications, another protein required for this interaction or a redox-signal coming from the mitochondrial matrix or from the chloroplasts could play a crucial role for all observations made in *in vivo* systems such as isolated protoplasts, or in yeast cells where the tested interactions take place in the nucleus. In order to study the redox-dependence of the observed interactions under defined conditions, various *in vitro* assays were performed with the purified recombinant proteins.

#### Co-sedimentation assays with rabbit F-actin and the glycolytic enzymes

**Figure 5:**
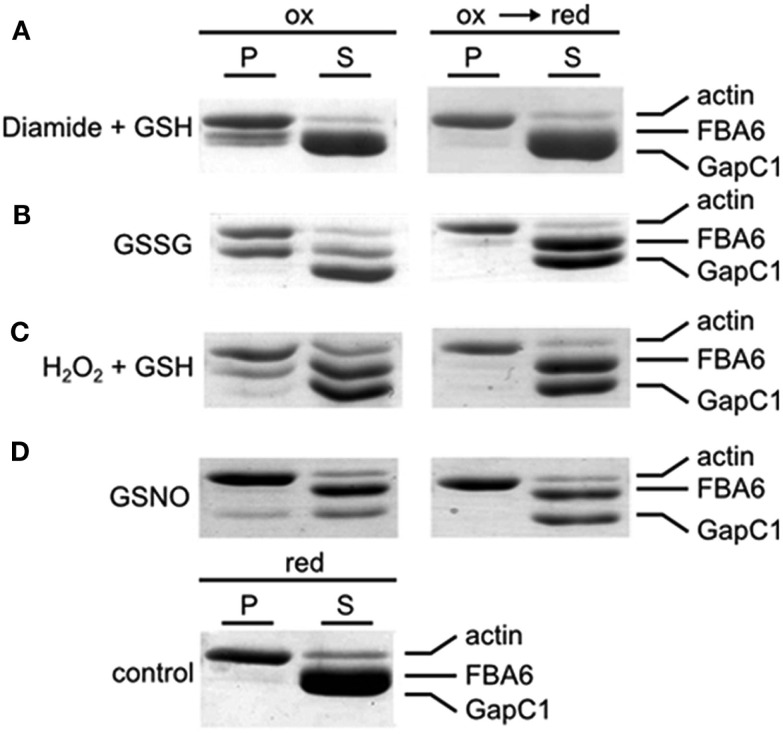
**Co-sedimentation assay with F-actin, GAPDH, and aldolase under oxidizing and reducing conditions**. Samples with rabbit F-actin, FBA6, and GapC1 were first oxidized with the specific redox reagents**(A)** Diamide + GSH (each 0.5 mM); **(B)** GSSG (5 mM);**(C)** H_2_O_2_ + GSH (each 0.5 mM); **(D)** GSNO (0.5 mM) with a following reduction by DTT_red_ (10 mM). The control sample with F-actin, FBA6, and GapC1 was only incubated with DTT_red_ (10 mM). The mixtures were then centrifugated at 100,000 × *g* for 1 h. Supernatant (S) and pellet (P) were separated for analysis by SDS-PAGE and the gels were stained with Coomassie Brilliant blue.

Incubation of F-actin with recombinant plant aldolase and GapC1 under reducing conditions and subsequent centrifugation resulted in a pellet fraction containing only actin and no glycolytic enzymes as can be seen from the SDS-PAGE analysis after ultracentrifugation (Figure 5, control). In contrast, incubation under various different oxidizing conditions resulted in the appearance of a certain portion of GapC and/or aldolase in the pellet fractions, diamide and GSSG treatment yielding mainly aldolase in the pellet, while GSNO resulted in GapC associating with F-actin. Subsequent reduction of an aliquot reversed the binding completely (Figure 5). Both, aldolase and GapC were also added separately to the F-actin preparation, yielding the same results as were obtained with both enzymes present simultaneously (data not shown).

#### Bundling assays with rabbit actin stained with phalloidin-rhodamin

**Figure 6:**
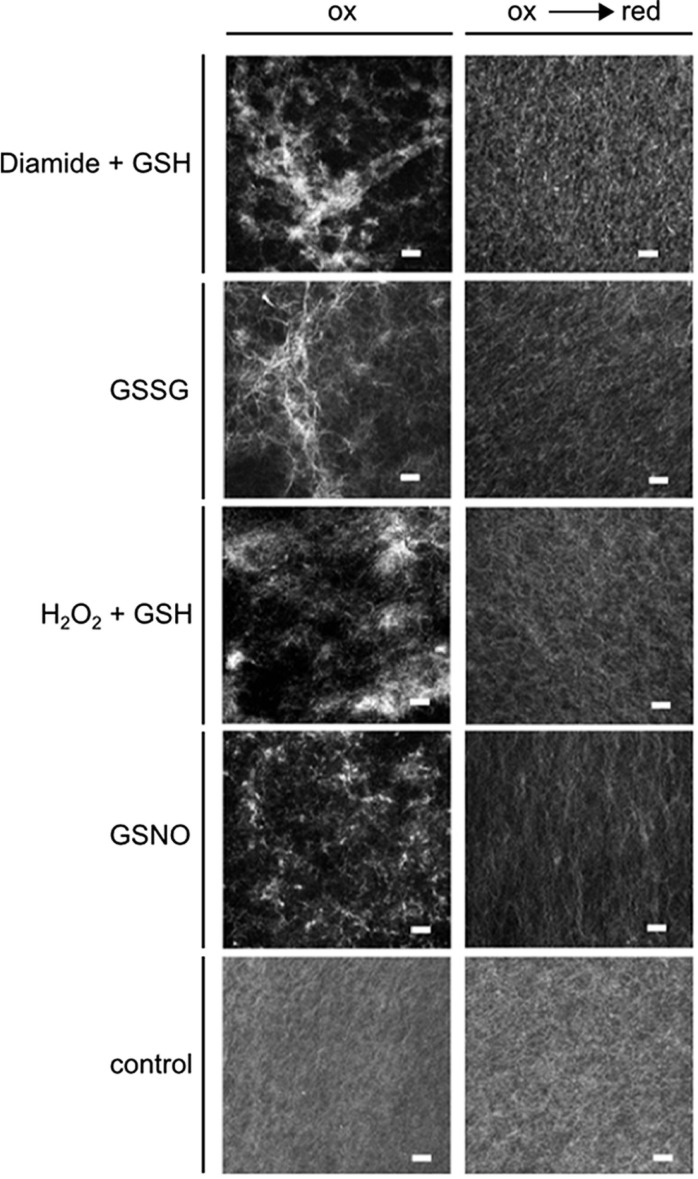
**Effect of redox-state on F-actin bundling activity of GapC and aldolase**. Samples with rabbit F-actin, aldolase, and GapC1 were first oxidized with the specific redox reagents: diamide + GSH (each 0.5 mM); GSSG (5 mM); H_2_O_2_ + GSH (each 0.5 mM); GSNO (0.5 mM) followed by a reduction step with DTT_red_ (10 mM). Subsequently, the samples were stained with rhodamine-labeled phalloidin (70 nM) and fluorescence was visualized by cLSM. The control experiment represents an incubation of F-actin with BSA incubated with diamide + GSH, and the subsequent reduction with DTT. Scale bar: 10 μm.

Bundle-like structures, formed by stained rabbit F-actin, were detected only under oxidizing conditions in the simultaneous presence of the glycolytic enzymes (Figure 6, left column), or of each of the enzymes alone (data not shown). The bundle structures disappeared when the sample was reduced subsequently with DTT (Figure 6, right column). When the enzymes were added in the presence of DTT, the same picture was apparent as with subsequent reduction of the previously oxidized sample (data not shown). In case, when only DTT was present without any enzymes added, there was no formation of bundles visible either (data not shown). In a control experiment, no glycolytic enzyme, but BSA was present instead, both under oxidizing and subsequent reducing conditions, and no bundle structures were visible under both conditions (Figure 6, lowest row). Although in these experiments it is not possible to detect any bound proteins, the bundling activity of the glycolytic enzymes became apparent, due to the changed appearance of the actin structure, as visualized by microscopy. Taken together, these experiments indicate that the glycolytic enzymes might act as bundling agents, but in a reversible manner only under oxidizing conditions.

#### Dot-blot overlay confirms redox-dependent binding of the glycolytic enzymes to F-actin and mitochondrial porin

**Figure 7:**
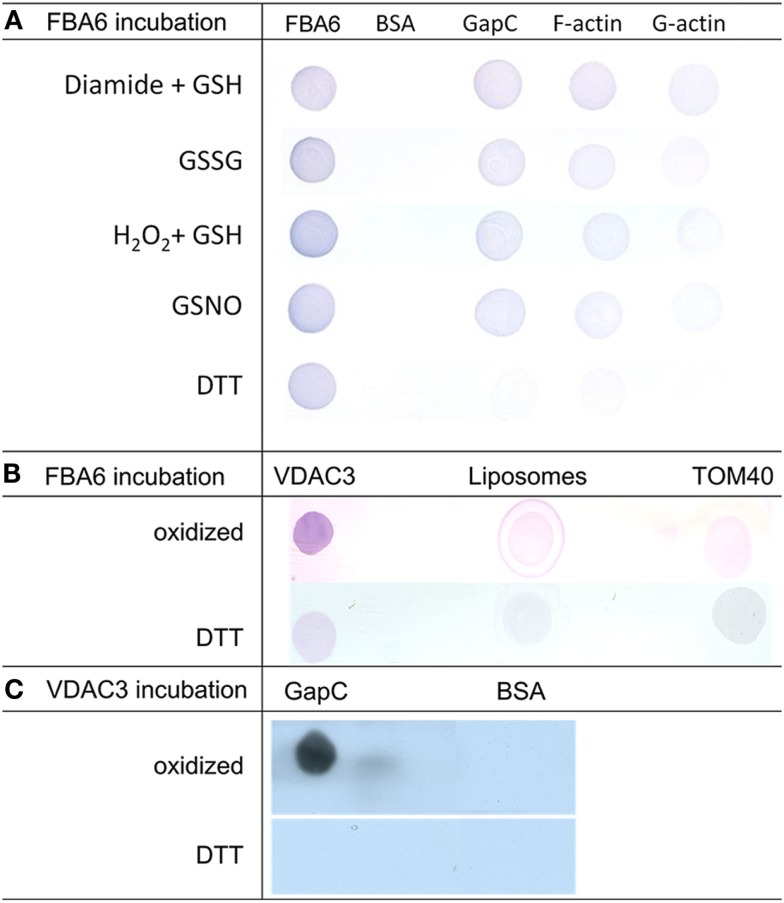
**Analysis redox-dependent binding of aldolase to GAPDH, actin and VDAC3 by Far Western blotting**. **(A)** Aldolase (FBA6; At2g36460), GAPDH (GapC1; At3g04120), G-actin, rabbit F-actin, and bovine serum albumin (BSA) as negative control (2 μg each) were applied onto a nitrocellulose membrane, overlaid with 100 μg/ml aldolase in the presence of 10 mM DTT, 0.1 mM GSNO, 5 mM GSSG, 1 mM diamide plus, 1 mM GSH, or 0.5 mM H_2_O_2_ plus 0.5 mM GSH, and probed with polyclonal antibody against maize aldolase as described in Section Experimental Procedures. **(B)** VDAC3 (At5g15090) reconstituted into liposomes was applied onto a nitrocellulose membrane, overlaid with 100 μg/ml aldolase in the presence of 10 mM DTT and without reductant (oxidized). Empty liposomes and TOM40 were used as controls, immunological detection of bound aldolase was as in **(A)**. **(C)**. GapC and BSA as a control were applied to the membrane, which was overlaid with VDAC3 in liposomes. Detection was with anti-VDAC1 serum and the HRP-conjugated second antiserum using ECL as a substrate as described in Section Experimental Procedures.

Redox-dependent binding of aldolase to F-actin, G-actin, and GapC1 under oxidizing conditions as observed in the co-sedimentation assays (Figure 5) was confirmed by the dot-blot overlay assay. Under all oxidizing conditions, a positive reaction was observed except with BSA that was used as a negative control. In the final immunodecoration any aldolase that had bound to a spotted protein could be detected (Figure 7). Glutathionylation, as well as nitrosylation resulted in binding of aldolase to F-actin, but also to GapC with a similar affinity. The interaction between G-actin and the modified FBA6 was much weaker than with the filamentous protein. Reduction prevented any binding of the added aldolase, only the formation of a homomeric oligomer of aldolase took place also under reducing conditions as apparent after immunostaining (Figure 7A, left lane).

The regulation of the direct binding of FBA6 and GapC to VDAC3 found in protoplasts was verified by means of a dot-blot overlay approach. Under oxidizing conditions the binding of aldolase to VDAC3 could be detected by immunodecoration with antibody against aldolase (Figure 7B). Similarly, a positive signal was also observed when oxidized GapC was overlaid with VDAC3 and immunodecorated with anti-VDAC antibody (Figure 7C). These positive reactions were not observed with the empty liposomes and TOM40 or with BSA, which were used as negative controls in this assay. Reduction prevented any binding of the glycolytic enzymes to the porin.

### Homology modeling of actin-binding

**Figure 8:**
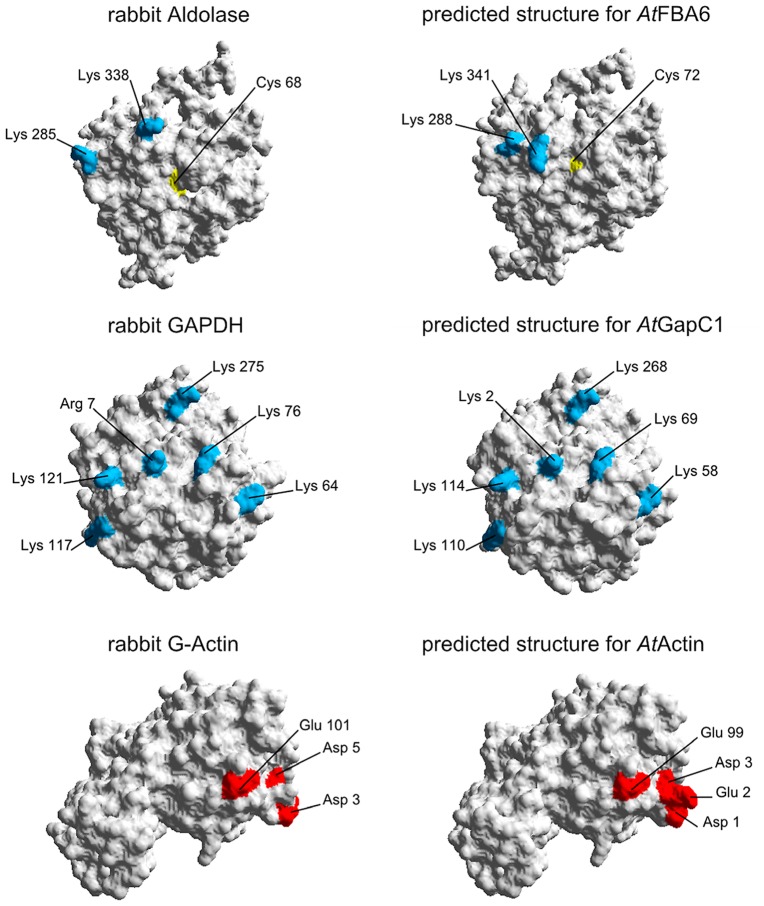
**Homology modeling of 3D-structures of aldolase, GAPDH, and actin**. On the left hand side, the proteins from rabbit as used by Forlemu et al. (2011) are shown. The corresponding *Arabidopsis* proteins on the right hand side were modeled with SWISS-MODEL as described in Section Experimental Procedures. By using the Swiss-Pdb Viewer 4.0.4, the molecular surface of the generated protein models was calculated and the conserved amino acids which are necessary for the protein–protein interaction are colored: Positively charged amino acids are blue, negatively charged amino acids are red. Cysteines are shown in yellow.

There is evidence from earlier work on the animal model that actin-binding sites contain amino-acid motifs with clustered charged residues, namely Asp and Glu, as well as Lys and Arg (Forlemu et al., 2011). It is obvious from the comparison with the animal counterparts that the positions of the positively and negatively charged residues on the surface of the proteins, which are implicated to be involved in the binding, are rather conserved, as are these proteins altogether (Figure 8). From the side view that is depicted to demonstrate the relative positions of these charged residues, it is not possible to locate surface Cys residues that have been shown to be subject to redox-modifications. However, it can be imagined that even distant molecular changes upon redox-modification of these residues can allosterically affect the binding sites by changing the charge distribution in the critical surface area.

## Discussion

### Binding of glycolytic enzymes to mitochondria

It appears to be a general phenomenon for cytoplasmic, metabolic proteins that organize into extensive physical structures upon nutrient depletion. Such functional foci formation may enhance substrate channeling and metabolite flux control, and decrease the free diffusion of intermediates (Ovadi and Saks, 2004). Moreover, a spatial or functional organization of metabolic pathways into complexes could flexibly assemble or disassemble upon demand of the cell. The enzyme–enzyme interactions may evoke locally high substrate concentrations and, in consequence, an enhanced transfer or metabolic channeling of intermediates from one active site of an enzyme to another (Ovadi and Srere, 1996). GFP-fused Ade4, a purine biosynthetic enzyme, expressed in yeast, formed foci in the absence of adenine and occurred in a diffused form when adenine was added (Narayanaswamy et al., 2009). The authors reported also on glutamine synthetase (Gln1) foci that cycled reversibly in the absence and presence of glucose. Such metabolon formation may also regulate competition between branched pathways for common metabolites, coordinate the activities of pathways with shared enzymes or intermediates, and sequester reactive or toxic molecules (Winkel, 2004). The observed dynamic association of FBA6-GapC complexes with mitochondria may respond to the respiration demands of the organelles, as suggested by Giegé et al. (2003). The authors proposed that attachment of the glycolytic reaction sequence to the outside of mitochondria may ensure the provision of a localized supply of pyruvate, in order to directly support mitochondrial respiration. Graham et al. (2007) demonstrated an evidence for physiological dependency for mitochondrial association of the glycolytic pathway. With the exception of hexokinase, the partitioning of all the glycolytic enzymes to the isolated *Arabidopsis* and potato tuber mitochondria decreased upon KCN treatment, which inhibits Complex IV of the mitochondrial respiratory chain. Stimulation of respiration with CCCP (carbonyl cyanide m-chlorophenylhydrazone), which uncouples the mitochondrial electron transport from ATP synthesis, led to an increase in the association of glycolytic enzymes with these organelles. A similar result was shown by Garmier et al. (2008) who reported on GAPDH (At3g04120) and aldolase (At3g52930) which, among several proteins analyzed with DIGE, decreased their abundance in isolated mitochondria following their treatment with rotenone, the inhibitor of Complex I of the mitochondrial respiratory chain. Our findings support therefore the concept of dynamic compartmentation of glycolytic metabolons, which in turn could enable a faster response of plant metabolism to environmental and physiological changes, reflected by variation in the cytosolic redox status.

Using GapC1 and GapC2 as baits in the yeast two-hybrid screen of the cDNA library from *Arabidopsis* suspension cell cultures gave initial hints for a direct association of these enzymes with the OMM via At5g15090-encoded VDAC3. This result was in good agreement with Graham et al. (2007), who succeeded in purifying GAPDH (At1g13440) and, in addition, the At5g15090-encoded VDAC as aldolase-interaction partners in a pull-down assay with the aldolase antibody. The authors suggested that GapC can attach at the surface of mitochondria directly via VDAC or indirectly, through an interaction with aldolase. Voltage-dependent anion channel (VDAC) is a porin-type β-barrel diffusion pore, located in the OMM (Clausen et al., 2004). This channel is functionally highly conserved among vertebrates, invertebrates, fungi, and plants, but the conservation is not as evident in the primary sequence, as it is in the secondary structure deduced from that sequence (Song and Colombini, 1996; Colombini, 2004). Our identification of VDAC3 as a GapC-interaction partner in the yeast two-hybrid screen and its confirmation *in vivo* via split YFP seemed to support the idea of a direct mitochondrial localization of GapC. Surprisingly, subsequent “one-on-one” yeast two-hybrid assays, where a single yeast strain (AH109) was cotransformed with two constructs encoding bait (GapC) and prey proteins (VDAC3), shed new light on the putative interaction. Due to the autoactivation of all reporter genes by the VDAC3, the interaction between GapC isoforms and the OMM porin emerged as a so-called false positive result (Figure 3). This could be caused by lack of certain posttranslational modifications in yeast cells or misfolding of the plant fusion protein that potentially may alter activity or binding of the investigated proteins. Therefore, it is often proposed to switch from the tested AD- to BD-fusion protein (Solmaz, 2003). According to the manufacturer, the pGADT7 vector runs expression of fusion proteins under a strong, full-length ADH1 promoter, in contrast to pACT2 or pGBKT7. This could lead to a high level of the VDAC3-AD fusion protein in the yeast cell and its autoactivating property. Interestingly, VDAC3 expressed from pACT2 also seemed to behave unspecifically in the described experiment, although the ADH1 promoter in this vector is truncated. The autoactivation property could be possibly explained by improper folding of this hydrophobic membrane protein under certain conditions prevailing in the yeast nucleoplasm.

### The role of VDAC-binding proteins in integrating metabolism and cellular functions

Holtgräwe et al. (2005) screened the cDNA library from hypoxic seedlings of *Zea mays* with the cytosolic isoform of maize aldolase and isolated the mitochondrial porin VDAC1a. The authors supported these findings by a dot-blot overlay assay, suggesting an interaction of the glycolytic enzyme with mitochondria via membrane porins. With a similar approach we found that the *in vitro* interaction of FBA6 or GapC with VDAC3 is stronger, when the glycolytic enzymes are not reduced with DTT (Figure 7). In plants, GAPDH appears to possess a general role in stress perception due to its reactive thiol, thus possibly functioning as a sensor for increased H_2_O_2_ levels (Hancock et al., 2006). The mitochondrial binding of aldolase or GapC seems therefore to be redox-dependent and connected to stresses that result in redox imbalance and generation of ROS and RNS in plant cells (see Leister, 2012). Previously, oxidative modifications and concomitant inactivation of both glycolytic enzymes had been demonstrated in *in vitro* experiments (Holtgrefe et al., 2008; van der Linde et al., 2011). Through association to the mitochondrial porin in their less active or even inactive form, the glycolytic enzymes might play an alternative function by carrying a redox-signal from cytosol to mitochondria and thus possibly initiating alternative responses, in extreme cases even cell death. In this context, it is noteworthy that the interaction of GAPDH with VDAC had been shown to be inhibited by DTT treatment in human cell lines (Tarze et al., 2007). In yeast, a central role of VDAC in transducing the cellular redox-state to the nucleus has been suggested (Galganska et al., 2010). In mammals, physical binding of GAPDH to VDAC1 was suggested to induce mitochondrial membrane permeabilization and apoptosis (Tarze et al., 2007). The association of the glycolytic enzymes with mitochondrial VDAC might well be connected with initial events during induction of programmed cell death (PCD).

VDAC facilitates metabolite exchange between the organelle and the cytosol, with higher affinity to organic anions, due to its highly conducting state, referred to as the open state (Colombini, 2004). VDAC is permeable to Ca^2+^ and regulated by different ligands, such as glutamate, ATP, or NADH (Colombini, 2004; Yehezkel et al., 2006), or by interacting proteins, such as actin (Xu et al., 2001), tubulin (Rostovtseva et al., 2008), or hexokinase (HXK; Kim et al., 2006; Balasubramanian et al., 2007). It also plays an active role in apoptosis by suppressing the release of apoptogenic factors into the cytosol, such as cytochrome *c* (Blachly-Dyson and Forte, 2001; Arzoine et al., 2009). It was demonstrated in mammalian cell lines that hexokinase can inhibit apoptosis by binding to VDAC (Azoulay-Zohar et al., 2004). Moreover, the respective cytoplasmic domains that are required for interaction with hexokinase and are involved in protection against cell death via inhibiting release of cytochrome *c*, were also found in the VDAC protein (Abu-Hamad et al., 2008). In *Arabidopsis* plants, interruption of hexokinase function activated PCD, whereas overexpression of predominantly mitochondria-associated HXK1 and HXK2 conferred enhanced resistance against H_2_O_2_ and α-picolinic acid (Kim et al., 2006). Interestingly, it was shown recently that not abiotic stress, such as drought, cold, salinity, but only bacterial pathogen infection leads to up-regulation of the expression level of four *Arabidopsis* VDACs (Lee et al., 2009). It becomes obvious that the VDAC-binding proteins are responsible for integration of plant metabolism and cellular functions, which might be also the case for aldolase and GapC.

### Redox-depedent binding of glycolytic enzymes to the actin cytoskeleton *in vitro*

Plants generate a series of signaling molecules, that may act in controlling processes such as growth, development, response to biotic and abiotic environmental stimuli, and PCD. These molecules are Reactive Oxygen Species (ROS), for instance, which result from a turnover of oxygen by cells of aerobic organisms, but in excess may cause damage to membrane lipids, DNA and proteins (Klatt and Lamas, 2000). ROS include singlet oxygen, H_2_O_2_, superoxide and hydroxyl radicals that remain under control, as long as the cellular antioxidant mechanisms comprising the radical-scavenging enzymes or redox-active compounds, such as the cysteine-containing tripeptide glutathione (GSH) and ascorbate, are functional (for review see: Foyer and Noctor, 2011). The cellular redox status depends on the ratio of the oxoidized and reduced forms of an intracellular pool of redox molecules, mainly GSH. Usually, GSH is present in millimolar concentrations and in up to 100-fold excess over GSSG. The oxidation of only a small amount of GSH to GSSG may dramatically change this ratio and, in consequence, the redox status of the cell. This, in turn, may evoke protein mixed disulfide formation or even protein degradation (Klatt and Lamas, 2000). In the cytoplasm, there are many redox-sensitive proteins that form transient disulfide bonds while catalyzing the reduction of thiol groups (Cumming et al., 2004). Glutaredoxins use the abundant GSH to reduce disulfide bonds via a thiol-disulfide interchange. The reduced thioredoxins belong to the second group of antioxidant enzymes that bind to substrate proteins containing a disulfide bond, while a dithiol-disulfide exchange reaction occurs, in which the active site cysteine residues of thioredoxin are oxidized, whereas the cysteine residues in the substrate protein are reduced (Cumming et al., 2004). In a situation, when the cellular redox homeostasis, i.e., the balance between prooxidants and antioxidants, is altered because of excessive production of ROS and/or impairment of cellular antioxidant mechanisms, cytosolic cysteine residues may become susceptible to oxidation. Under non-stressed conditions, disulfide bond formation occurs primarily in the oxidizing environment of the ER in eukaryotic cells, and in chloroplasts upon darkening.

Along with ROS, Reactive Nitrogen Species (RNS) are also produced in plant cells. While the role of NO as a signaling molecule is better understood, less information is available on other RNS, but it is well known, that analogous damage due to RNS during the nitrosative stress may take place in the plant cell upon high salinity, for instance (Valderrama et al., 2007). *S*-nitrosylation of plant proteins was demonstrated by treating extracts from *Arabidopsis* cell suspension cultures with the NO-donor *S*-nitrosoglutathione (GSNO) and by exposure of plants to gaseous NO (Lindermayr et al., 2005). Among over 50 *S*-nitrosylated proteins, stress-related, redox-related, signaling/regulating, cytoskeleton, as well as metabolic proteins were detected in both approaches. With respect to the present study, interesting *S*-nitrosothionylated targets were tubulin α and β, actin isoform ACT2/7, and the glycolytic enzymes GAPDH, aldolase, triose-P isomerase, phosphoglycerate kinase, and enolase. Interestingly, S-glutathionylation of both GapC isoforms, as well as aldolase, enolase, sucrose synthase, and cytoskeletal components – ACT2/7, tubulin α and β – were also shown to occur in *Arabidopsis* suspension cultures upon treatment with the oxidant tert-butylhydroperoxide (Dixon et al., 2005). NO is assumed to play a role in signaling also in plants (Moreau et al., 2010). Moreover, NO signaling has been proposed to involve posttranslational modification of cytoskeletal elements in many plant stress response and developmental processes (Yemets et al., 2011). The experimental increase of NO levels in cells of maize roots was shown to reversibly impact the actin cytoskeleton assembly and its organization (Kasprowicz et al., 2009). ROS signals were also implicated in actin reorganization and PCD in pollen tubes during self-incompatibility response (Wilkins et al., 2011). NO is also known as a multi-faceted signaling molecule that acts in many cellular processes, such as stomatal closure, seed germination, root development, senescence, flowering time, activation of defense-related genes, and hypersensitive cell death (Wang et al., 2006).

Posttranslational modifications are of great interest for the protein–protein interaction events. Hara et al. (2005), for instance, demonstrated that before human GAPDH reaches the nucleus, it undergoes *S*-nitrosylation, which triggers its interaction with Siah1 and enables the nuclear translocation of GAPDH. In response to environmental fluctuations and stressors, the complex regulation of metabolic enzymes may function through posttranslational modifications that can affect enzymatic activity, intracellular localization, protein–protein interactions, and stability (Huber and Hardin, 2004). Therefore, a new issue of stress-induced covalent modifications of the enzyme appear to be crucial for the microcompartmentation events that are involved in the regulation of many cellular processes, in particular in pathophysiology (Marozkina and Gaston, 2012). Interestingly, NO might have also impact on the actin protein directly or on actin-binding proteins (Kasprowicz et al., 2009). In fact, upon nitrosylation with GSNO, GapC was found in a pellet of filamentous rabbit actin, but when it was reduced with DTT, it did not interact with F-actin and remained soluble (Figure 5, fourth lane). Under these conditions, FBA6 was not interacting with F-actin, as if there were a competition for a binding site at F-actin. Moreover, nitrosylated, as well as glutathionylated GapC and FBA6 together caused bundling of actin filaments that was reversible upon reduction (Figure 6). The incorporation of NO, as well as glutathione upon formation of a mixed disulfide could be shown for *Arabidopsis* GapC (Holtgrefe et al., 2008) and aldolase (van der Linde et al., 2011). The reactive cysteine residues in the active site of both *Arabidopsis* GapC1 and GapC2 isoforms were shown by Holtgrefe et al. (2008) as susceptible to thiol modification and oxidation. A similar bahaviour was described for cytosolic aldolase isoforms (van der Linde et al., 2011). The addition of GSSG and GSNO was shown to inactivate these enzymes, and this inactivation was fully or at least partially reversible upon addition of DTT. These results indicate that GapC and aldolase might overtake a new role as actin bundling proteins in their inactive form. Since Schmitz and Bereiter-Hahn (2002) did not find any correlation in cytoskeletal association of human GAPDH in presence and absence of NO, they proposed that the association of GAPDH to stress fibers in human cells deprived of serum is unlikely to have a general function, such as to create a glycolytic microcompartment or to allow an enhanced glycolytic flux via metabolic channeling (Knull and Walsh, 1992; Al-Habori, 1995; Masters, 1996). Instead, Schmitz and Bereiter-Hahn (2002) suggested that the cytoskeletal association of GAPDH upon serum depletion might serve initiating cytoskeletal rearrangements during apoptosis, which is induced by prolonged serum withdrawal. In plants, there is also evidence that rearrangement of the cytoskeleton is actively involved in signaling the need for PCD, not only as a consequence of this event occurring under stress and during development (for review see: Smertenko and Franklin-Tong, 2011). A binding and bundling function is conceivable for *Arabidopsis* GapC and aldolase upon oxidizing conditions, occurring when plants are exposed to biotic or abiotic stress. Regulation of the cytoskeletal reorganization upon binding of GapC and aldolase in plants remains to be elucidated in future investigations.

### Actin-binding region in aldolase and GAPDH

The actin-binding sites of aldolase and GAPDH have been investigated in the animal model using peptide binding and site-directed mutagenesis (Humphreys et al., 1986; O’Reilly and Clarke, 1993; Wang et al., 1996), as well as by a Brownian Dynamics approach which underlines the ionic nature of these interactions (Forlemu et al., 2011). It seems indeed that the contact sites of the interactions carry a high number of charged amino acids, which occur in clusters over the whole sequences of actin (mainly negatively charged Glu and Asp residues), aldolase (Lys residues at the C-terminus), and GAPDH (both positively and negatively charged Lys, Glu, and Asp residues highly accumulating in the N-terminal half of the sequence; Forlemu et al., 2011). Since actin and the glycolytic enzymes are highly conserved in all organisms, the same pattern of charge distribution at largely conserved positions is apparent in the *Arabidopsis* homologs (Figure 8). Therefore, it can be assumed that binding of aldolase and GapC1 in *Arabidopsis* takes place in a similar manner. Taking into account the Cys modifications that lead to inactivation of cytosolic aldolase (van der Linde et al., 2011) and GAPDH (Holtgrefe et al., 2008), the intensity of the interaction might well be influenced, resulting in the differential binding properties described here. The S-glutathionylation of Cys-374 (Dalle-Donne et al., 2003) and the disulfide formation between Cys-285 and Cys-374 of actin (Farah and Amberg, 2007) have been implicated in being an essential part of oxidative stress sensing in human and yeast cells, respectively. In proteomic analyses to identify potentially *S*-nitrosylated plant proteins, among many others also GAPDH, aldolase, and actin were identified (Lindermayr et al., 2005). The importance of posttranslational modifications to carefully orchestrate protein–protein interactions in larger protein ensembles, by influencing the strength of the interactions within a functional module, has been put forward by Stein et al. (2009). Such dynamic interactions are of particular importance in transient microcompartments, as occurring in signaling processes, e.g., initiated by NO (Marozkina and Gaston, 2012). Future work with mutated protein constructs, with either Cys residues or charges modified in the critical regions of the proteins, will allow to study the effect of changed surface properties on the affinities *in vitro* and *in vivo*.

### Predictions from databases

Many databases are available with results from analyses with respect to putative protein–protein interactions in all organisms. A recent attempt has been made to compile all this information concerning the cellular networks for *A. thaliana* in one single database, thus combining knowledge coming from many different, experimental approaches, by creating the interactive web tool ANAP (*Arabidopsis* Network Analysis Pipeline; Wang et al., 2012) and similar databases. ANAP compiles more than 200,000 interaction pairs from the various sources, whereby a large number of the interactions are assumed from coexpression data. Only in some of the cases, biochemical investigations have followed up such putative interactions. From this database, we have extracted information concerning the candidates in focus in the present study (Table 1). Not in all cases interactions were found for the identical isoforms, then also some closely related isoforms are included into the table. But the overall picture underlines the interactions between aldolase, GAPDH, VDAC, and actin investigated here. Data from databases (source for evidence is specified by the letters A–E) in combination with our experimental *in vivo* and *in vitro* findings (shaded in gray) suggest a model, where the transient formation of a contact between mitochondria (via VDAC) and the actin cytoskeleton is achieved by interactions with the glycolytic enzymes aldolase and GAPDH. The dynamic rearrangement in the cytosol is thought to occur upon redox-changes as part of a signal transduction chain.

**Table 1:** **Predicted and experimentally verified interactions between aldolase, GAPDH, actin and VDAC**.

	ACT8	FBA4	FBA6	GapC1	VDAC1	VDAC2	VDAC3
ACT8			A	A	A		
FBA4			B, C, D	B, C			
FBA6	A	B, C, D		A, B, C	A	A	A
GapC1	A	B, C	A, B, C		A	A	A
VDAC1	A		A	A		A	A, C, E
VDAC2			A	A	A		
VDAC3			A	A	A, C, E		

*The ANAP network analysis tool was used to compile evidences for interactions deduced from various types of experiments or setups. A, Coexpression; B, Inferred by curator; C, Interologs mapping; D, Phylogenetic profile; E, Predicted text mining. The gray background indicates interaction partners confirmed experimentally in this work*.

## Conclusions and Future Perspectives

Actin dynamics and redox-changes are involved in many stress and developmental processes (for review see: Yemets et al., 2011). Therefore, the restructuring of the actin cytoskeleton, association of VDAC-binding proteins at mitochondria, possibly leading to changed functions, such as induction of PCD, and the transfer of the glycolytic enzymes to the nucleus, are potential steps in a complex signal transduction network (for review see: Scheibe and Dietz, 2012). The term “moonlighting” reflects the phenomenon of independent, non-catalytic functions that a well known catalytic protein may possess additionally, due to separate functional domains (Moore, 2004). These multiple functions could be attained as a consequence of changes in the cellular localization of a protein, its expression by different cell types, its oligomeric state, or the cellular concentration of a ligand, substrate, cofactor or product (Jeffery, 1999). Novel unexpected features and variable subcellular locations have already been revealed for the mammalian GAPDH (Hara et al., 2006; Sirover, 2011; Tristan et al., 2011). In plants, the phenomenon of moonlighting functions of the glycolytic enzymes is yet to be investigated in more detail. As redox-changes are now discovered to occur – at least transiently – in all cell compartments in all organisms, any redox-challenge imposed on the cell can be transformed into a specific thiol modification pattern, finally leading to the required response and to acclimation (Spadaro et al., 2010). It is, however, likely that this dynamic level of regulation, as suggested here by the observations *in vivo* and *in vitro*, is even more important in photoautotrophic organisms where the conditions fluctuate dramatically (Potters et al., 2010; Scheibe and Dietz, 2012). In view of this fact, our study aims at the understanding of a dynamic protein interaction network that might be involved in retrograde redox-signal processing.

## Conflict of Interest Statement

The authors declare that the research was conducted in the absence of any commercial or financial relationships that could be construed as a potential conflict of interest.

## Abbreviations

ACT, actin; BiFC, bimolecular fluorescence complementation; CFP, cyan fluorescent protein; cLSM, confocal laser scanning microscopy; diamide, azodicarboxylic acid bis(dimethylamide); DTT, dithiotreitol; ER, endoplasmatic reticulum; FBA, cytosolic fructose 1,6-bisphosphate aldolase; GapC/GAPDH, cytosolic NAD-dependent glyceraldehyde-3-phosphate dehydrogenase; GFP, green fluorescent protein; GSH/GSSG/GSNO, reduced/oxidized glutathione/nitrosoglutathione; HXK, hexokinase; OMM, outer mitochondrial membrane; PM, plasma membrane; VDAC, voltage-dependent anion channel; YFP, yellow fluorescent protein.
